# Relationship Between Patient Satisfaction and the Presence of Cats in Psychiatric Wards

**DOI:** 10.1089/acm.2018.0263

**Published:** 2018-12-14

**Authors:** Julia C. Templin, Karin Hediger, Cora Wagner, Undine E. Lang

**Affiliations:** ^1^ University Psychiatric Clinic (UPK), University of Basel, Basel, Switzerland.; ^2^ Faculty of Psychology, Department of Clinical Psychology and Psychotherapy, University of Basel, Basel, Switzerland.; ^3^ Swiss Tropical and Public Health Institute, Basel, Switzerland.; ^4^ Institute for Interdisciplinary Research on Human-Animal Relationship (IEMT), Basel, Switzerland.

**Keywords:** patient satisfaction, psychiatric therapy, animal-assisted intervention, human-animal interaction, cats

## Introduction

The presence of animals in psychiatric clinics is thought to positively influence patients' satisfaction. Whereas psychiatric clinics often house animals on their premises, (i.e., 60% in Germany)^1^ there are no studies on the effects of free unstructured contact between patients and these animals. We investigated the relationship between the presence of cats in psychiatric wards and patient satisfaction of patients with depression, substance abuse, or depression in stationary psychiatric treatment.

## Methods

We retrospectively analyzed available data from 170 in-patients treated in 2016 at the University Psychiatric Clinic (UPK) in Basel, Switzerland, with a diagnosis of psychosis, depression, or substance abuse in certain wards. The selected wards were organized in a comparable format, but either housed a cat or did not for each diagnosis group. Patients were allocated to the wards according to their diagnoses, but randomly with regard to the presence of ward cats. Patients in wards with cats could freely interact with the cats in the wards at any time. Patient satisfaction was assessed by self-report at the end of the treatment with the 27-item “Münsterlinger Patientenfragebogen” (MüPF27).^2^ Group differences between patients in wards with and without cats were analyzed in SPSS Statistics, using the Mann–Whitney *U*-test with a significance level of *p* ≤ 0.05.

## Results

Among the 170 patients in this study, the mean age was 51.3 years (SD = 15.5), 16 nationalities were represented, 46.50% were women, 44.1% had depression, 25.9% had substance abuse, and 30% had psychosis as a main diagnosis.

Patients living in wards with a cat had significantly higher overall satisfaction than patients living in a ward without a cat (Table 1). Patients living in the presence of a cat were also more satisfied with their treatment outcome and recommended the clinic more. Moreover, they rated their recreational opportunities, the common rooms, and the collaboration with their primary nurse, social worker, other therapists, and psychologists significantly better, whereas there was no effect regarding the collaboration with the doctor. Patient groups in wards with and without cats did not differ regarding their satisfaction with their rooms, the food, and the cafeteria (Table 1).

**Table 1.** Group Differences

*MüPF item*	*Condition*	N	*M*	*SD*	z	p	*Cohen's* d
Overall satisfaction	With cat	81	5.70	1.55	−2.25	0.024^a^	0.34
	Without cat	86	5.14	1.78			
Treatment outcome	With cat	78	5.82	1.13	−3.40	0.001^a^	0.53
	Without cat	82	5.30	0.95			
Recommendation of the clinic	With cat	78	5.91	1.54	−2.27	0.023^a^	0.35
	Without cat	83	5.31	1.87			
Common rooms	With cat	80	5.44	1.79	−1.99	0.047^a^	0.31
	Without cat	84	5.06	1.62			
Leisure opportunities	With cat	77	5.30	1.84	−2.64	0.008^a^	0.42
	Without cat	81	4.53	1.93			
Collaboration with primary nurse	With cat	80	6.36	1.19	−2.67	0.008^a^	0.38
	Without cat	83	5.82	1.63			
Collaboration with social worker	With cat	50	6.02	1.48	−2.11	0.035^a^	0.39
	Without cat	58	5.19	2.11			
Collaboration with further therapists	With cat	72	6.28	1.27	−2.96	0.003^a^	0.46
	Without cat	77	5.58	1.79			
Collaboration with psychologist	With cat	71	6.18	1.32	−2.01	0.044^a^	0.34
	Without cat	45	5.42	2.01			
Collaboration with doctor	With cat	79	5.97	1.49	−1.55	0.121	0.23
	Without cat	81	5.73	1.52			
Patient's room	With cat	81	5.42	2.02	−0.97	0.331	0.12
	Without cat	86	5.84	1.58			
Food	With cat	81	5.28	1.86	−0.37	0.708	0.06
	Without cat	86	5.13	1.96			
Cafeteria	With cat	71	5.62	1.61	−1.22	0.221	0.20
	Without cat	78	5.31	1.69			

^a^ Statistically significant.

M, mean; MüPF27, 27-item Münsterlinger Patientenfragebogen; *N*, number of patients; SD, standard deviation; z, *Z*-score.

## Discussion

Psychiatric inpatients were significantly more satisfied with their stay at the clinic when their ward housed a cat. The observed small-to-medium effects have the potential to be clinically meaningful. Previously, the presence of animals had been shown to create a more comforting clinic environment,^3^ and to lead to a more positive perception of other people and rooms.^4^ The fact that there was no effect on satisfaction with the patient's own rooms, the food, or the cafeteria would also support this hypothesis since cats were not allowed in the patient's rooms, the dining rooms, or the cafeteria. The higher satisfaction with the collaboration with their primary nurse, their social worker, their other therapists, and their psychologists is in line with previous research reporting that people's trust and therapeutic alliance can be enhanced in the presence of a dog.^5–7^ However, we found no relationship between the presence of a cat and the patient's perceived collaboration with doctors. This highlights the importance of future work that examines the causal role of an animal's presence on such measures.

Limitations of the study include that the retrospective cross-sectional design does not allow for causal conclusions, and that no additional measures of psychiatric illness severity, exposure to and attitudes toward the cat, and possible negative effects were available. Future studies are needed to investigate possible effects of the presence of animals in psychiatric clinics.
